# Mitochondrial Localization of the Yeast Forkhead Factor Hcm1

**DOI:** 10.3390/ijms21249574

**Published:** 2020-12-16

**Authors:** María José Rodríguez Colman, Joaquim Ros, Elisa Cabiscol

**Affiliations:** Departament de Ciències Mèdiques Bàsiques, IRBLleida, Universitat de Lleida, 25198 Lleida, Spain; M.J.RodriguezColman@umcutrecht.nl (M.J.R.C.); Joaquim.ros@udl.cat (J.R.)

**Keywords:** forkhead transcription factor, Hcm1, mitochondria, cell cycle, mitochondrial metabolism, mtDNA, yeast

## Abstract

Hcm1 is a member of the forkhead transcription factor family involved in segregation, spindle pole dynamics, and budding in *Saccharomyces cerevisiae*. Our group described the role of Hcm1 in mitochondrial biogenesis and stress resistance, and in the cellular adaptation to mitochondrial respiratory metabolism when nutrients decrease. Regulation of Hcm1 activity occurs at the protein level, subcellular localization, and transcriptional activity. Here we report that the amount of protein increased in the G1/S transition phase when the factor accumulated in the nucleus. In the G2/M phases, the Hcm1 amount decreased, and it was translocated outside the nucleus with a network-like localization. Preparation of highly purified mitochondria by a sucrose gradient density demonstrated that Hcm1 colocalized with mitochondrial markers, inducing expression of *COX1*, a mitochondrial encoded subunit of cytochrome oxidase, in the G2/M phases. Taken together, these results show a new localization of Hcm1 and suggest that it acts as a mitochondrial transcription factor regulating the metabolism of this organelle.

## 1. Introduction

To ensure the optimum adaptation of cells to environmental changes, a network of complex signaling pathways has evolved. Transcription factors play essential roles in controlling multiple cellular processes through gene expression regulation. In mammals, forkhead transcription factors (FKH-TFs) respond to a wide range of external stimuli such as nutrients and hormones like insulin, growth factors, neurotrophins, cytokines, and several stresses, including oxidative stress. FoxO transcription factors (FoxO1, 3, 4, and 6), a subfamily of FKH-TFs, control various biological functions including stress resistance, DNA repair, metabolism, cell cycle, and aging (reviewed in Refs. [1,2,3]). In accordance with such a variety of functions, their activity is regulated by phosphorylation, acetylation, ubiquitination, methylation, and glycosylation. A combination of these post-translational modifications controls FoxO protein levels, DNA-binding properties, and subcellular localization [3,4,5].

*Saccharomyces cerevisiae* has four members of the forkhead family of eukaryotic transcription factors (Fkh1, Fkh2, Fhl1, and Hcm1), classified on the basis of a conserved DNA-binding domain [6]. Hcm1 was first identified as a high copy suppressor of a defect in spindle pole assembly [7]. This cell-cycle-specific transcription factor was initially described to regulate the transcription of genes involved in chromosome organization, spindle dynamics, and budding [8]. It is periodically transcribed and functions as an S-phase-specific transcriptional activator [9]. Our group described that Hcm1 is also involved in mitochondrial biogenesis and stress resistance [10], and it plays a role in adaptation to respiratory metabolism in response to early nutrient limitation [11]. Moreover, it was found that Hcm1 controls life span independently of calorie restriction [12]. During aging, a rapid nuclear exclusion of Hcm1 leads to vacuolar alkalization and replicative senescence [13]. Regulation of Hcm1 activity occurs at several levels: gene expression, protein–protein interaction, post-translational modification, degradation, and subcellular localization [9,10,11,13,14,15].

Hcm1 interacts with Sir2, an NAD+-dependent histone deacetylase. In response to oxidative stress stimuli, sirtuin activity increases Hcm1 nuclear accumulation and, therefore, Hcm1-mediated gene expression involved in mitochondrial metabolism and oxidative stress resistance [10]. In fact, Fkh1 and Fkh2, whose expression is activated by Hcm1 [9], are also associated with Sir2 and coordinate a protective response against stress [16]. Hcm1 reacts both to glucose and nitrogen deficiency, participating in the metabolic shift from fermentation to respiration. In this context, the main kinases involved in nutrient response pathways (Snf1, Tor1, and Sch9) regulate Hcm1 transcriptional activity, with phosphorylation by Snf1 having a preponderant role [11].

In mammals, FoxO3 has been localized inside the nucleus, cytoplasm, as well as in the mitochondrial matrix and the mitochondrial outer membrane [17,18]. In yeast, Hcm1 was the only FKH-TF found outside the nucleus. The localization of Hcm1 in both the cytoplasm and nucleus was mentioned in a large-scale analysis of protein localization in budding yeast [19] and was also observed in previous studies from our group [10].

In this study, we report a new localization of the yeast Hcm1 in the mitochondria. In the G2/M phases, GFP-labeled Hcm1 from synchronized cells showed a network-like structure outside the nucleus. Highly purified mitochondria reveal that this transcription factor colocalizes with mitochondrial markers, in agreement with the pattern observed by microscopy. Moreover, the mitochondrial encoded gene *COX1* displays an Hcm1-dependent pattern with maximum expression in G2/M phases, supporting the role of Hcm1 as a mitochondrial transcription factor involved in activation of mitochondrial activity. These results are in line with Hcm1 acting as a nuclear regulator of mitochondrial respiration as previously described [10,11].

## 2. Results and Discussion

### 2.1. Cell Cycle Regulates Hcm1 Levels and Localization

**Figure 1:**
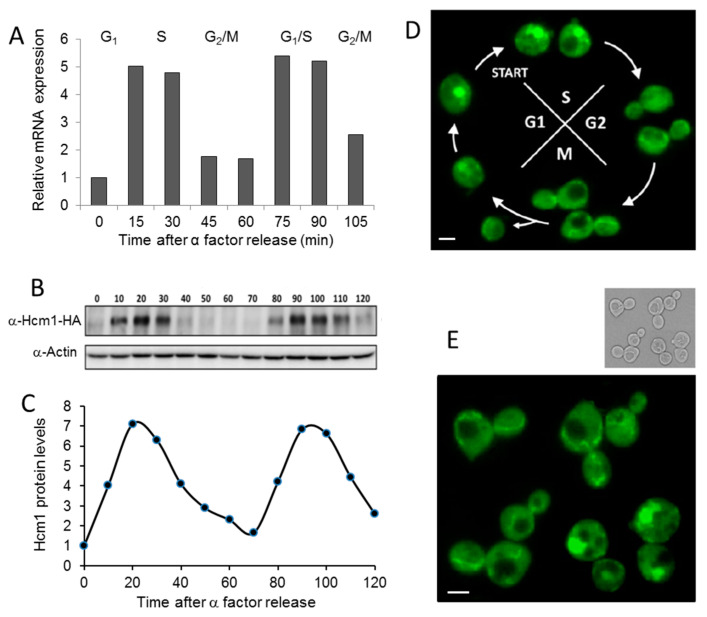
Cell cycle modulates the amount of Hcm1 and its localization. Cells grown exponentially were synchronized to G1 with α-factor for 2 h, and several parameters were determined at different times after release. (**A**) Hcm1 mRNA expression of WT (*HCM1-HA*) cells was determined by quantitative real-time PCR analysis. Actin expression was used as an internal control to normalize expression levels. (**B**) HA-tagged Hcm1 was analyzed by Western blot with anti-HA antibodies. Actin was used as a loading control. (**C**) Bands corresponding to Hcm1 in B were quantified, normalized by actin levels, and the relative intensities were calculated. (**D**) Pictures by fluorescence microscopy of *HCM1-GFP* cells were taken at different time points of the cell cycle. (**E**) Amplified fluorescent microscopy picture of several *HCM1-GFP* cells synchronized in the M phase. Scale bar, 1 µm.

It is known that the yeast forkhead transcription factor *HCM1* is periodically transcribed during the cell cycle [9]. In addition to *HCM1* expression, we studied Hcm1 protein levels and localization through different phases of the cell cycle using alpha factor to synchronize the culture. Alpha factor stops the cell cycle at the end of the G1 phase. After release, cells resume growth synchronically [10]. Figure 1A,B shows that both mRNA and protein levels were highly regulated by the cell cycle, with maximum amounts in the late G1 and early S phase (Figure 1C) when the transcription factor was concentrated in the nucleus (Figure 1D). Inside the nucleus it regulates the transcription of genes involved in chromosome segregation. Genes regulated by Hcm1 peak primarily during the late S phase [9]. Although chromosome segregation is impaired in the absence of Hcm1, budding kinetics under optimal growth conditions in *Δhcm1* cells is quite similar to wild-type cells [9]. In addition to this classical role, Hcm1 also participates in the adaptation of cells from fermentative to respiratory metabolism during nutrient scarcity. Hcm1 phosphorylation by Snf1 (sucrose-nonfermenting kinase), the founding member of the AMPK family, results in a shift from the cytoplasm to the nucleus and increased transcriptional activity of genes involved in respiration, use of alternative energy sources, NAD synthesis, and oxidative stress resistance [11]. Using GFP-labeled Hcm1 can reveal that, besides Hcm1 nuclear localization during the G1/S phases, Hcm1 also appeared with a network-like localization, which is clearly observed in the M phase (Figure 1E). Such a network structure resembles the mitochondrial organization observed in yeast [16].

### 2.2. Mitochondrial Localization of Hcm1

**Figure 2:**
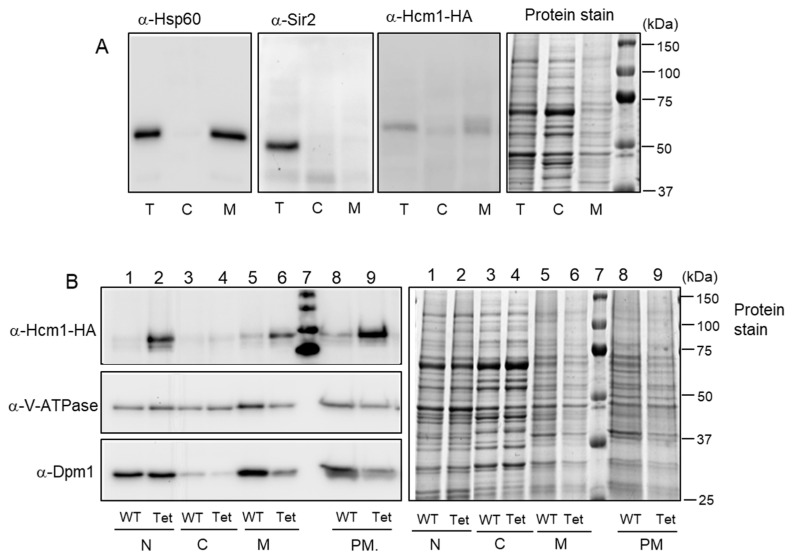
Analysis of Hcm1 localization by cell fractionation followed by sucrose-gradient centrifugation. (**A**) WT (*HCM1-HA*) cells growing exponentially were analyzed by Western blot of Hsp60 (mitochondrial protein), Sir2 (nuclear protein), and Hcm1 from total extracts and cytosolic and mitochondrial-enriched fractions. (**B**) WT (*HCM1-HA*) and Hcm1-overexpressing (*tetHCM1-HA*, labeled as tet) cells growing exponentially were fractionated in nuclear, cytosolic, and mitochondrial- enriched fractions (lanes 1 to 6). Mitochondrial-enriched fractions were subjected to a sucrose gradient centrifugation to obtain purified mitochondria (lanes 8 and 9). All samples were analyzed by Western blot with anti-HA (Hcm1), V-ATPase (vacuolar ATPase), and Dpm1 (ER dolichol-phosphate mannosyltransferase subunit 1 protein). Protein stain with Brilliant Coomassie Blue is shown as a loading control. T: total cell extract, C: cytosolic fraction, M: mitochondrial fraction, N: nuclear fraction, PM: purified mitochondria.

Having in mind that Hcm1 promotes mitochondrial biogenesis and is involved in oxidative metabolism [10], we explored the possibility that Hcm1 could shift from the nucleus to mitochondria in the G2/M phases. To this end, a cell extract fractionation was prepared to separate the cytosolic fraction from the mitochondrial-enriched fraction using exponentially grown WT (*HCM1-HA*) cells. Total, cytosolic, and mitochondrial-enriched fractions were analyzed by Western blot to detect Hcm1 localization. A mitochondrial protein (Hsp60) and a nuclear protein (Sir2) were also detected using specific antibodies and compared to Hcm1 (Figure 2A). Hsp60 appeared, as expected, in the total cell extract and in the mitochondrial-enriched fraction, with a pattern similar to that of Hcm1. As predicted, Sir2 only appeared in the total cell extract, containing the nuclei. Because endogenous levels of Hcm1 are very low (see Ref. [10] to compare with other yeast forkheads like Fkh1, Fkh2, and Fhl1), an Hcm1-overexpressing strain was used. *TetHCM1-HA* cells were grown and cell extract fractionation was performed by differential centrifugation, as described in Materials and Methods. In this experiment, nuclear, cytosolic, and mitochondrial-enriched fractions were obtained. Figure 2B (lanes 1 to 6) shows Western blot images of anti-Hcm1, anti-V-ATPase, and anti-Dpm1. WT cells were submitted to the same procedure and used as a control (Figure 2B). In addition, to attain a sample with highly purified mitochondria, the mitochondrial-enriched fraction previously obtained was submitted to ultracentrifugation with a sucrose density gradient. Mitochondrial fractions were pooled, and proteins were detected by Western blot (Figure 2B, lanes 8 and 9).

**Figure 3:**
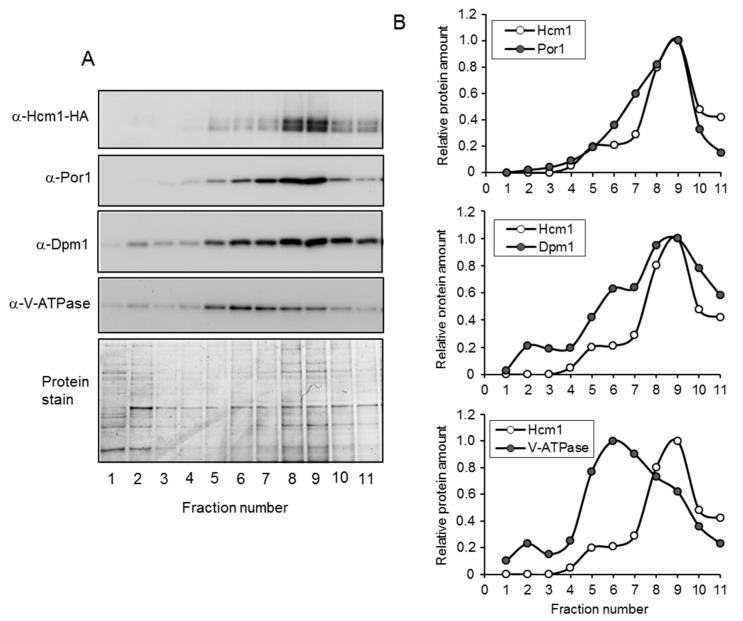
Fraction analysis of the sucrose gradient centrifugation used in mitochondrial purification. (**A**) Mitochondrial-enriched fractions from *tetHCM1-HA* cells were separated by four-phase sucrose gradient centrifugation. The distribution of Hcm1 was analyzed in each fraction by Western blot with anti-HA. Western blot anti-porin (mitochondria), anti-V-ATPase (vacuole), and anti-Dpm1 (ER) were used as markers of these organelles. Fractions 1 to 11 represent the top and the bottom of the sucrose density gradient, respectively. Protein stain with Brilliant Coomassie Blue is shown as a loading control. (**B**) Bands corresponding to Hcm1, porin, V-TPase, and Dpm1 in A were quantified. In each image, the fraction with the highest value was normalized to 1.

Overexpression of Hcm1 clearly showed that this transcription factor localized in the nucleus and also in the mitochondria. However, even highly purified mitochondria was contaminated with vacuolar and ER components. This is not unusual, since such structures are very difficult to fully split from mitochondria. To solve this problem and establish whether Hcm1 was localized inside the mitochondria or associated with other contaminant organelles, the profile of this protein was analyzed through the total sucrose gradient and compared with the profiles of V-ATPase, Dpm1, and Por1. To that end, the mitochondrial-enriched fraction was loaded on top of a four-phase sucrose gradient (60%, 32%, 23%, and 15%) and centrifuged at 134,000× *g* for 60 min. All gradient was collected in 11 fractions. Proteins were separated by SDS-PAGE and Hcm1 plus the marker proteins, detected by Western blot (Figure 3A). Bands were quantified from each fraction, and the relative amounts are shown in Figure 3B. The vacuolar marker, V-ATPase, was detected throughout the entire gradient, with enrichment in fractions 5 to 9. Dpm1, the ER marker, was also detected along the whole gradient, with a maximum from fractions 6 to 11. In the case of the mitochondrial marker Por1, it could be noticed starting at fraction 4 and enriched from fractions 6 to 10. As can be seen in Figure 3B, the Hcm1 profile closely resembled that of mitochondrial Por1. The Hcm1 profile did not match with the vacuolar marker or the ER marker, although differences with Dpm1 were not as clear as with V-ATPase.

V-ATPase is encoded by the *VMA2* gene and is the β subunit of the eight subunits that constitute the membrane domain of V-ATPase. This protein acts as a proton pump located in the cellular endomembrane system [20], but its localization is mostly vacuolar [21]. On the other hand, Dpm1 corresponds to a dolichol mannose phosphate synthetase. This enzyme exerts its activity on the cytosolic face of the endoplasmic reticulum (ER), and the modified sugar enters the lumen of the reticulum [22]. Like the vacuolar marker (V-ATPase), Dpm1 is also a membrane protein. The porin Por1 was used as a mitochondrial marker. Por1 is a voltage-gated anion channel that is located in the mitochondrial outer membrane. This protein participates in the osmotic stability and permeability of the mitochondria [23,24].

It is common for cell homogenization to result in the rupture of large structures such as the ER and perhaps also vacuoles. This rupture may generate fragments of membranes that would reassemble into structures with density similar to mitochondria and, as a consequence, co-purify with this organelle. In addition, it must be considered that the ER is part of the yeast endomembrane system that surrounds many organelles and encompasses a large portion of the cell [25]. Thus, it seems unlikely that mitochondria could be exclusively isolated without dragging any membranous portion of the reticulum. Taken together, these results highly suggest that after nuclear exclusion in the G2/M phases, Hcm1 showed a mitochondrial localization with a network-like structure. However, the possibility that Hcm1 could be located in mitochondria-associated membranes (MAM), a region of the ER reversibly tethered to mitochondria, cannot be completely dismissed.

### 2.3. Hcm1, mtDNA Copy Number, and Mitochondrial Gene Activation

**Figure 4:**
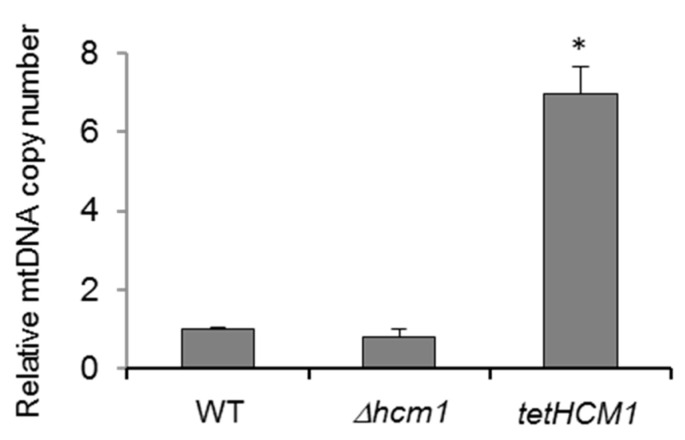
Hcm1 and mtDNA copy number. The number of copies of mtDNA was normalized by the number of copies of nDNA as described in Materials and Methods. *COX1* was used as a target mitochondrial gene, and *ACT1* was used as the reference nuclear gene. Gene amplification was carried out by real-time qPCR in a duplex reaction from total DNA from the indicated strains. Statistical analysis was performed comparing mutants with WT cells. The data are represented as the means ± SD from at least three independent experiments. * *p* < 0.01.

Hcm1 is involved in mitochondrial metabolism, since cells overexpressing Hcm1 display increased levels of mitochondrial proteins and oxygen consumption [10]. To obtain further evidence, the mitochondrial DNA (mtDNA) copy number was measured. mtDNA is quantified by the ratio between a target mitochondrial gene and a reference nuclear gene (mtDNA/nDNA) using quantitative real-time PCR. To that end, total DNA was used to amplify the mitochondrial *COX1* gene and the nuclear *ACT1* gene in a simultaneous reaction. As shown in Figure 4, the mtDNA copy number was much higher in *tetHCM1-HA* cells and slightly lower (but not statistically significant) in *Δhcm1* cells, compared to WT cells. Thus, Hcm1 levels correlated to mitochondrial abundance. 

**Figure 5:**
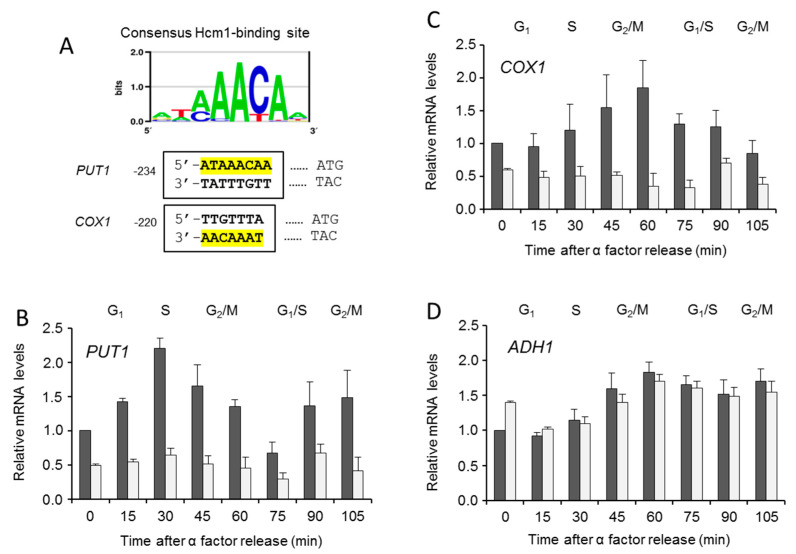
Hcm1-dependent gene expression and cell cycle. (**A**) Consensus binding motifs for Hcm1 in the promoter regions of *PUT1* and *COX1*. *COX1* motif was found on the Crick strand (http://yetfasco.ccbr.utoronto.ca) [26]. WT and *Δhcm1* cells grown exponentially were synchronized to G1 with α-factor for 2 h, and mRNA of genes were determined by quantitative real-time PCR analysis at different times after release. (**B**) mRNA expression of *PUT1*, a nuclear encoded gene. (**C**) mRNA expression of *COX1*, a mitochondrial encoded gene. (**D**) mRNA expression of *ADH1*, a nuclear encoded gene used as a control. The data are represented as the means ± SD from at least three independent experiments.

However, such results did not directly imply a role of Hcm1 inside the mitochondria. To explore further, expressions of two genes throughout the cell cycle were measured in WT and *Δhcm1* synchronized cells. They were *PUT1*, a nuclear gene known to be directly regulated by Hcm1 [10], and *COX1*, a mitochondrial gene with a potential Hcm1-binding site [9] (Figure 5A). *PUT1*, coding for the enzyme proline oxidase, is needed to use proline in the mitochondria as an energy source when nutrients are scarce [11]. The *COX1* gene encodes the Cox1 subunit, which, together with Cox2 and Cox3, compose the core of the cytochrome oxidase complex, the last electron acceptor in the mitochondrial respiratory chain. *PUT1* and *COX1* showed a cell-cycle-dependent expression pattern, which was abolished in *Δhcm1* cells (Figure 5B,C). As a negative control, we evaluated the expression of *ADH1*, a nuclear gene coding for the cytosolic enzyme alcohol dehydrogenase 1 with no Hcm1-binding site in its promoter. As expected, *ADH1* expression was unaffected in *Δhcm1* cells (Figure 5D). Interestingly, the expression profiles of the *PUT1* and *COX1* genes were almost opposite. *PUT1* followed a pattern similar to Hcm1 that was slightly delayed in time (compare Figure 5B with Figure 5C). This is in agreement with the nuclear localization of Hcm1 and its role as a transcription activator of nuclear genes [9,11]. The expression profile of the mitochondrial *COX1* showed a maximum in the G2/M phases (Figure 5C), when Hcm1 was found outside the nucleus. Such a specific pattern of *COX1* expression that is Hcm1-dependent and coincides with the mitochondrial localization of Hcm1 supports the role of Hcm1 acting as a mitochondrial transcription factor. This role would be similar to that described for mammalian FoxO3. A low-glucose regimen induces the formation of a FoxO3A, SirT3, and mitochondrial RNA polymerase complex, causing activation of the mitochondrial genome and a subsequent increase in mitochondrial respiration [13]. Likewise, in metabolically stressed cancer cells, ERK and AMPK activation induce mitochondrial translocation of FoxO3 and expression of the mitochondrial genome to support mitochondrial metabolism [18]. In this condition, FoxO3 was also localized in the mitochondrial outer membrane. Although we cannot rule out Hcm1 being associated with the mitochondria or any membranous structure associated with mitochondria, the fact that mitochondrial localization in the G2/M phases coincided with *COX1* gene expression reinforces the role of Hcm1 as a mitochondrial transcriptional activator.

In conclusion, our results point, for the first time, at the mitochondrial localization of Hcm1. Furthermore, our data suggest that Hcm1 translocates from nucleus to mitochondria in the G2/M phases and induces expression of mitochondrial encoded genes, such as *COX1*, favoring mitochondrial metabolism. Although further functional studies are required to fully elucidate the mitochondrial role of Hcm1, these results are in accordance with Hcm1 acting in coordination both as a mitochondrial and nuclear activator involved in mitochondrial respiratory metabolism and stress resistance. They also define Hcm1 as a key player to adapt yeast cells to nutrient scarcity.

## 3. Materials and Methods

### 3.1. Yeast Strains and Growth Conditions

*S. cerevisiae* strains used in this work are listed in Supplementary Table S1. All strains employed in this work derived from CML128 (*MATa ura3-52 his4 leu2-3,112 trp1*) [27]. A HA-tagged Hcm1 strain, *HCM1-HA*, was used as a WT strain. For microscopic studies, a GFP-tagged Hcm1 strain was used. HA and GFP were C-terminally labeled [10]. Null mutant *Δhcm1* was obtained by using the short flanking homology approach after PCR amplification of the *natMX4* cassette. Disruption was confirmed by PCR analysis. Overexpression of the Hcm1 protein *TetHCM1-HA* was obtained by replacing the endogenous promoter with a tetO_7_ promoter as described [28]. The cells were grown at 30 °C by incubation in a rotary shaker at 180 rpm using YPD medium (1% yeast extract, 2% peptone, 2% glucose).

### 3.2. Cell Extracts Fractionation and Mitochondria Purification

To obtain mitochondrial enriched fractions, yeast cells (2 OD) grown in YPD to an A_600_ = 1 were centrifuged and resuspended with 100 mM Tris-HCl pH 9.4, 10 mM DTT. After 20 min incubation at 30 °C, cells were centrifuged at 3000× *g* for 5 min, and the supernatant was discarded. Cells were washed in a 20 mM sodium phosphate buffer, pH 7.4, 1.2 M sorbitol, and resuspended in the same buffer plus zymolyase to remove the cell wall. After 30–45 min incubation at 30 °C, spheroplast were centrifuged at 3000× *g* for 5 min, washed in 20 mM sodium phosphate buffer, pH 7.4, 1.2 M sorbitol, and resuspended in 10 mM Tris-HCl pH 7.4, 0.6 M sorbitol, 1 mM EDTA, 0.2% (*w*/*v*) BSA, and protease inhibitors. Spheroplasts were homogenized with a Dounce homogenizer (Sigma-Aldrich, Madrid, Spain) and centrifuged at 4000× *g* for 5 min. Nucleus and cell debris were pelleted, and the supernatant solution containing the cytosolic plus the mitochondrial fraction was centrifuged again at 12,000× *g* for 15 min. The pellet containing the enriched mitochondrial fraction was resuspended in a 10 mM MOPS-KOH buffer pH 7.2, 250 mM sucrose, 1 mM EDTA. Samples were either used as such for Western blot or used for further purification to obtain highly purified mitochondria.

Highly pure fractions of mitochondria were obtained as described [29] with some modifications. In brief, a four-phase sucrose gradient (60%, 32%, 23%, and 15%) was prepared in an ultracentrifuge tube (total volume of 4.5 mL). From 100 to 500 µL of enriched mitochondrial fraction (at 5 mg/mL) was carefully loaded on top of the gradient and centrifuged at 134,000× *g* for 60 min at 4 °C. All gradient was collected in 500 µL fractions, and each fraction was diluted with 1 mL of 10 mM MOPS-KOH buffer pH 7.2, 250 mM sucrose, 1 mM EDTA. Mitochondria and other organelles were pelleted by centrifugation at 10,000× *g* and resuspended in 32 µL of 10 mM MOPS-KOH buffer pH 7.2, 250 mM sucrose, 1 mM EDTA plus 8 µL of loading buffer (375 mM Tris-HCl pH 6.5, 10% SDS, 25% β-mercaptoethanol, 50% glycerol, 6 mM bromophenol blue). Samples were finally incubated for 5 min at 95 °C before SDS-PAGE.

### 3.3. Yeast Cell Synchronization

The cells were synchronized by a block and release protocol (α-factor treatment). The cultures were grown to an A_600_ = 0.75 in YPD, and α-factor (Gene Script Biotech, Leiden, The Netherlands, ref RP01002) was added to a concentration of 5 µg/mL. After 45 min, α-factor was added again to a final concentration of 10 µg/mL. After 90 min, synchronized cells in G1 were checked by visualization of nonbudded cells by contrast microscopy. Alpha-factor was removed by pelleting the cells for 4 min at 4000 rpm at 4 °C. The cells were washed once with cold YPD. G1-arrested cells were resuspended in fresh YPD when indicated. Samples were taken every 15 min for gene expression analysis by qPCR.

### 3.4. Gene Expression Analysis

Quantitative real-time PCR (RT-PCR) was performed using the TaqMan System (Applied Biosystems, Thermo Fisher Scientific, Waltham, MA, USA) [30]. Total RNA was extracted using the RNeasy kit (Qiagen, Barcelona, Spain ref. 74104) according to the manufacturer’s instructions, and 1 μg total RNA from each sample was converted into cDNA with 50 ng utilized for each individual RT-PCR assay in a 48-cycle, three-step PCR reaction using the iCycler (Bio-Rad, Alcobendas, Madrid, Spain). Amplification was performed using the TaqMan Universal PCR Master Mix kit (Applied Biosystems, Thermo Fisher Scientific, Waltham, MA, USA, Cat. 4304437). Quantification was completed using iCycler IQ Real-Time detection system software (version 2.3, Bio-Rad, Alcobendas, Madrid, Spain). For all gene expression analyses, actin (*ACT1*) was used as an internal control. Data represent at least three technical repeats of each analysis.

An mtDNA copy number was normalized versus a nuclear DNA copy number. To this end, the *COX1* gene was amplified by real-time PCR as a measure of the mitochondrial DNA index, and the *ACT1* gene was amplified as the nuclear DNA index. Both amplifications were carried out at the same time in the same well because the Taqman probes are made with different fluorophores (VIC for *ACT1* and FAM for *COX1*) and can be detected in distant fluorescence channels. The reactions were carried out from total DNA, in quadruplicate and in a final volume of 20 µL. The reagents used were the same as described above. Statistical analysis was performed using the Student’s *t* test.

All RNAs were analyzed using predesigned Taqman Gene Expression Assays from Life Technologies (Thermo Fisher Scientific, Waltham, MA, USA). References: *HCM1*: Sc04105445_s1; *ADH1*: Sc04163599_s1; *PUT1*: Sc04147047_s1; *COX1*: Sc04164539_s1; *ACT1*: Sc04120488_s1.

### 3.5. Western Blot Analysis

Cell extracts were obtained as described [31], separated in SDS-PAGE, and transferred to polyvinylidene difluoride membranes (Immobilon-P, Millipore, Mollet del Vallès, Spain). Anti-HA (1:2500 dilution, from Roche, Sant Cugat, Spain or Biolegend, San Diego, CA, USA, ref. 1-867-423), anti-actin (1:2000 dilution, from Chemicon, Thermo Fisher Scientific, Waltham, MA, USA, ref. MAB1501R), anti-Hsp60 (1:6000 dilution, from StressGen, San Diego, CA, USA, ref. SPA808), anti-Sir2 (1:11,000 dilution, from Santa Cruz Biotechnology Inc., Heidelberg, Germany, ref. SC-6666), anti-Por1 (1:1000 dilution, from Molecular Probes, Thermo Fisher Scientific, Waltham, MA, USA, ref. A6449), anti-Dpm1 (1:250 dilution, from Invitrogen, Thermo Fisher Scientific, Waltham, MA, USA, ref. A6429), and anti V-ATPase (1:4000 dilution, from Invitrogen, Thermo Fisher Scientific, Waltham, MA, USA, ref. A6427) were used as primary antibodies. The secondary antibodies conjugated to horseradish peroxidase were used as follows: goat anti-mouse antibody (1:40,000 dilution, from Pierce, Thermo Fisher Scientific, Waltham, MA, USA, ref. 31430), goat anti-rat (1:4000 dilution, from Molecular Probes, Thermo Fisher Scientific, Waltham, MA, USA, ref. A10549), and rabbit anti-goat (1:25,000 dilution, from Thermo Fisher Scientific Sci., Waltham, MA, USA, ref. A10549). Images were acquired in a ChemiDoc XRS System (Bio-Rad, Alcobendas, Madrid, Spain) and analyzed with Quantity One software (Bio-Rad, Alcobendas, Madrid, Spain).

### 3.6. Microscopy Studies

At different times after α-factor synchronization, HCM1-GFP-labeled cells grown in YPD medium (0.5 ODs) were centrifuged at 3000× *g* for 3 min and resuspended in 40 µL of 20 mM sodium phosphate buffer, pH 7.4. Cells were analyzed by fluorescence microscopy (Olympus DP30 BW, L’Hospitalet de Llobregat, Spain) using 488 nm laser excitation for GFP, and pictures were taken. Fluorescent images were adjusted to better visualize localization.

## Supplementary Materials

Supplementary materials can be found at https://www.mdpi.com/1422-0067/21/24/9574/s1.

## Abbreviations

FKH-TF	Forkhead transcription factor
ER	Endoplasmic reticulum
mtDNA	Mitochondrial DNA
nDNA	Nuclear DNA
