# Supplementary material for: Mitochondrial Localization of the Yeast Forkhead Factor Hcm1
Source: Int J Mol Sci. 2020 Dec 16;21(24):9574. doi: 10.3390/ijms21249574 (PMC7765673; doi:10.3390/ijms21249574)
Supplement: Supplementary file 1 [file ijms-21-09574-s001.pdf]

Supplementary materials

Table S1. Yeast strains used in this work.

Strain	Relevant genotype	Comments	Source
CML128	*MAT*a *ura3-52 his4 leu2-3,112 trp1*	Wild type	[27]
GRB2405	CML128 *HCM1-3HA::natMX4*	Chromosomal *HCM1* tagged with 3HA using the *natMX4* cassette	[10]
MJRC07	GRB2405 *tetO$_7$-HCM1-3HA::kanMX4*	Integration of tetO7-regulatable *HCM1-HA* in GRB2405	[10]
MJRC05	CML128 *hcm1::natMX4*	HCM1 disruption with *natMX4* cassete	[10]
MJRC08	CML128 *HCM1-GFP:: kanMX4*	Chromosomal *HCM1* tagged with GFP using the *sGFP-ADH1t-kanMX4* cassette	[10]

[10] Rodriguez-Colman, M.J.; Reverter-Branchat, G.; Sorolla, M.A.; Tamarit, J.; Ros, J.; Cabiscol, E. The forkhead transcription factor Hcm1 promotes mitochondrial biogenesis and stress resistance in yeast. *J. Biol. Chem.* **2010**, *285*, 37092–37101, doi:10.1074/jbc.M110.174763.

[27] Gallego, C.; Garí, E.; Colomina, N.; Herrero, E.; Aldea, M. The Cln3 cyclin is down-regulated by translational repression and degradation during the G1 arrest caused by nitrogen deprivation in budding yeast. *EMBO J.* **1997**, *16*, 7196–7206, doi:10.1093/emboj/16.23.7196.
